# Detecting Moments of Stress from Measurements of Wearable Physiological Sensors

**DOI:** 10.3390/s19173805

**Published:** 2019-09-03

**Authors:** Kalliopi Kyriakou, Bernd Resch, Günther Sagl, Andreas Petutschnig, Christian Werner, David Niederseer, Michael Liedlgruber, Frank Wilhelm, Tess Osborne, Jessica Pykett

**Affiliations:** 1 Department of Geoinformatics, University of Salzburg, 5020 Salzburg, Austria; 2 Center for Geographic Analysis, Harvard University, Cambridge, MA 02138, USA; 3 Department of Cardiology, University Hospital Zurich, 8091 Zurich, Switzerland; 4 Department of Psychology, University of Salzburg, 5020 Salzburg, Austria; 5 Department of Demography, Faculty of Spatial Sciences, University of Groningen, PO Box 800, 9700 AV Groningen, The Netherlands; 6 School of Geography, Earth and Environmental Sciences, University of Birmingham, Birmingham B15 2TT, UK

**Keywords:** stress detection, rule-based algorithm, physiological wearable sensors, real-world field studies, perceived stress

## Abstract

There is a rich repertoire of methods for stress detection using various physiological signals and algorithms. However, there is still a gap in research efforts moving from laboratory studies to real-world settings. A small number of research has verified when a physiological response is a reaction to an extrinsic stimulus of the participant’s environment in real-world settings. Typically, physiological signals are correlated with the spatial characteristics of the physical environment, supported by video records or interviews. The present research aims to bridge the gap between laboratory settings and real-world field studies by introducing a new algorithm that leverages the capabilities of wearable physiological sensors to detect moments of stress (MOS). We propose a rule-based algorithm based on galvanic skin response and skin temperature, combing empirical findings with expert knowledge to ensure transferability between laboratory settings and real-world field studies. To verify our algorithm, we carried out a laboratory experiment to create a “gold standard” of physiological responses to stressors. We validated the algorithm in real-world field studies using a mixed-method approach by spatially correlating the participant’s perceived stress, geo-located questionnaires, and the corresponding real-world situation from the video. Results show that the algorithm detects MOS with 84% accuracy, showing high correlations between measured (by wearable sensors), reported (by questionnaires and eDiary entries), and recorded (by video) stress events. The urban stressors that were identified in the real-world studies originate from traffic congestion, dangerous driving situations, and crowded areas such as tourist attractions. The presented research can enhance stress detection in real life and may thus foster a better understanding of circumstances that bring about physiological stress in humans.

## 1. Introduction

In the discipline of psychobiology, stress is defined as a complex reaction consisting of physiological and psychological (i.e., cognitive, affective and behavioral) components [1]. It is considered an unpleasant emotional state that people experience in situations perceived as highly challenging or physically threatening [2,3]. The term was first introduced by Hans Selye, the “father of stress”, who noticed that patients with various diseases suffer physically from the same non-specific symptoms that constitute a response to a stimulus [4,5]. Walter Bradford Cannon introduced the concept of “fight or flight” to describe the phenomenon of how a body’s nervous system is activated when faced with a stressor, leading the body to release stress hormones for its protection [4,5,6]. The autonomic nervous system and hypothalamic-pituitary-adrenal (HPA) axis are two major systems that respond to stress as an attempt to re-establish homeostasis (a “steady state”) on a psychophysiological level [7,8,9]. This involves changes in cardiac activity, sweat gland activity, and skin temperature. Thus, physiological signals, including galvanic skin response and skin temperature, that are related to such activities, can provide insights into ANS activity [8,10] and are considered to be reliable indicators of stress [11]. This psychobiological account of stress is particularly useful in research that aims to locate specific extrinsic stressors at specific moments in space and time. The increasing availability of inexpensive and sophisticated measurement systems establishes the basis for novel research ideas to inform fundamental questions for emotion researches [12]. Various physiological parameters and parameter combinations can be utilized to detect stress [13]. Previous approaches indicate that the combination of several physiological signals does not ensure the highest possible accuracy. However, most researchers have used galvanic skin response, mainly combined with electrocardiogram or Blood volume pulse. Most importantly, the design details of each algorithm appear to play a crucial role.

Methodologically, the use of Support Vector Machine (SVM) dominates previous studies for stress detection. There are also studies that use machine learning algorithms or that introduce feature-based algorithms.

Numerous approaches for stress detection that use wearable physiological sensors have been described; many fall into one of two categories. The first is that researchers develop a method using laboratory data, but do not investigate the efficiency of the method in real-world studies. This means that the proposed method has limited validity outside the constraints of laboratory settings. On the other hand, several approaches have been developed based on real-world data, but their validity for detecting true psychological stress is hampered by limitations inherent in defining the ground truth in the assessment of stressful events. Typically, the approach is to correlate physiological signals with the spatial characteristics of the physical environment, which is sometimes supported by video recordings. To our knowledge, there are a few research efforts that have aimed at combining the two approaches and define and detect stress in a well-controlled laboratory context and apply and extend the gained knowledge in real-world settings [14,15,16].

Our aim is to leverage the capabilities of laboratory assessment and low-cost wearable physiological sensors to detect moments of stress (MOS) in real-world settings. We introduce an algorithm that advances a priori knowledge from laboratory experiments and acquires a posteriori knowledge in a real-world field study. This requires the use of low-cost wearable sensors in a minimally invasive mobile setting both in the laboratory and field. Thus, sensors with complex installation and calibration procedures or sensors that contain subjects’ activity (like wired sensors or sticky electrodes on the torso) cannot be used. Furthermore, eye-tracking devices are not suitable for investigating stress moments in real-world settings because they can be sensitive in their operation, failing to work effectively if the conditions are not controlled [17]. A further requirement of our particular research setting is the availability of a geospatial component (e.g., a GPS sensor) to geo-locate the detected MOS.

Thus, we propose a rule-based algorithm for stress detection based on galvanic skin response and skin temperature without using a machine-learning algorithm, as many researchers have done in the past. We use rule-based methods that allow us to integrate expert knowledge and contribute to the actual understanding of the investigated phenomenon. To do this, we created a framework of rules, weights, and critical values for stress detection based on previous approaches and an experimental process using laboratory data. We then carried out a laboratory experiment to collect data of physiological responses to a particular stress stimulus. This way, we created a “gold standard” of physiological responses to stressors based on which we developed and calibrated the algorithm. We further validated our algorithm in two real-world field studies in a mixed-methods approach through spatially correlating the test persons’ perceived stress assessed by questionnaire, the geo-location of questionnaire responding, and the corresponding real-world situation that was captured on head-mounted camera video recordings. This mixed-methods approach overcomes the shortcomings of every single method. Our results indicate that the algorithm is able to detect MOS with 84% accuracy, showing high correlations between physiologically measured (wearable sensors), self-reported (questionnaire), and recorded (video) stress events. Therefore, our approach goes beyond the current state of the art for stress detection by combining experimental with real-world processes and combining empirical data with expert knowledge.

## 2. Related Work

### 2.1. Physiological Parameters as Indicators of Stress

Galvanic skin response (GSR), also called skin conductance (SC) or electrodermal activity (EDA), is a biomarker of sympathetic nervous system activation [18] and is considered one of the most sensitive and valid markers of emotional arousal. During high levels of emotional arousal, sweat secretion is intensely activated, which can be measured using a GSR sensor accurately and easily on hands and feet [19]. There is a linear relationship between arousal and skin conductance up to very high levels where the skin gets saturated [7]. There are two types of skin conductance, i.e., the skin conductance level (SCL) and the skin conductance response (SCR). The SCL, or tonic skin conductance, is the baseline level of a recording during an experiment without any environmental events, while the SCR, or phasic skin conductance, represents the body’s reaction in the presence of a stimulus [20,21]. Various features of GSR can be used to investigate ANS activity, such as (1) amplitude of the response, i.e., the difference between the highest SCR occurring at the peak of the response and the pre-stimulus SCL [22]. It has been observed that a higher amplitude is caused by a more intense stimulus [20,23]. (2) Latency is the temporal distance between the stimulus and the start of the response. The average latency was found to be around 3 s [18], but it varies from 1 to 5 s in different studies [24,25,26]. However, high-intensity stimuli lead to shorter latencies [20,23]. (3) Rising time is the time between the onset of the stimulus and the peak of the response [22]. An intense stimulus affects the rising time [23] and may vary from 1 to 5 s. Shorter rising times tend to appear after longer latencies [7,27]. (4) Recovery time of the response is defined as the time from the peak value of GSR to the point of 50% recovery of the initial level before the stress event [28]. It is probable that the recovery will be asymptotical or a full recovery may not occur at all. For this reason, recovery time is measured from the peak to the point of 50% recovery [18]. The half-recovery time typically corresponds to approximately 1 to 10 s [27,29]. (5) The number of responses [30] and (6) the area under the curve is also used [7] in GSR analysis. (7) Response slope is the rate of the response over time [29]. Steeper slopes indicate a more intense stress moment [31,32]. According to our literature review, the most widely used GSR features are amplitude, rising time, and the number of responses, whereby the particular feature combination varies depending on the scope of the study.

Skin temperature (ST) usually varies from 32 to 35 °C [33]. However, in some cases, it may show greater variations because of extreme environmental temperature, fever, malnutrition, physical exertion, and physiological changes such as vasospasm [34]. Skin temperature can be easily and reliably measured using a temperature sensor in contact with the skin [35] and has been used in numerous studies for emotion detection. However, there is an ambiguity concerning the impact of stress on skin temperature. Some studies confirm that skin temperature rises in the presence of stress [36], while other studies find that skin temperature decreases under stress [1,10,37,38].

Heart rate variability (HRV) is another promising marker [39] that reflects the sympathetic and parasympathetic activities of the ANS measuring the temporal difference between successive heartbeats. The time between beats is measured in milliseconds and is called the R-R interval or Inter-beat interval (IBI). HRV is a commonly used feature in stress detection as it is considered a very sensitive indicator of stress [11,35,40]. There are parameters that are used to study the HRV in the time domain such as mean value, the standard deviation of RR intervals, root mean square, etc. [35]. In the frequency domain, the most widely used method is low frequency (LF), high frequency (HF), LF/HF ratio, etc. [41].

Numerous other physiological signals can be used for stress detection, such as the electrocardiogram (ECG or EKG), which records the electrical activity of the heart on the skin’s surface. Many researchers have used this physiological signal for stress detection. Electromyography (EMG) measures the muscles’ activity [42]. While some muscle contractions are under voluntary control, some are activated mostly involuntarily, including facial contractions in response to stressors [38]. Blood volume pulse (BVP) is the amount of blood in vessels during a certain time interval and is an indicator of blood flow. BVP decreases under stress due to vascular constriction and increases in a calm state [1]. Respiration rate (RESP) measures the speed of respiration of a person by, for instance, recording chest expansion through a resistor that is integrated into a chest belt and measures the impedance. Stress is typically characterised by an increased respiratory rate. Respiration can influence the ECG signal by causing a peak in the low frequencies of the ECG spectrogram. RESP is not highly correlated with stress [30]. The electroencephalogram (EEG) measures the alternating currents of ensembles of brain neurons using headsets [43].

### 2.2. Stress and Stress Level Detection

A literature review on stress detection strategies to investigate emotional states using physiological signals unveils a variety of approaches. There are studies attempting to detect whether a subject is stressed or not, such as [44] wherein participants were asked to solve arithmetic problems under time pressure and psychosocial stress induced by social-evaluative threat. They fed SVM with GSR features and achieved an accuracy of 82.8%. Zhai and Barreto [45] measured BVP, GSR, and ST in combination with pupil dilation using the Stroop Test as a stressor. Then they investigated the accuracy of various learning algorithms selecting the SVM as the most accurate (90.1%). In [46], they proposed an emotional stress recognition system based on GSR, EEG, BVP, and respiration rate. They used pictures of the International Affective Picture System (IAPS) to elicit stress and SVM as a classifier achieving the accuracy of 82.7%. The researcher in [47] presented a study of stress identification based on Heart Rate (HR), HRV, GSR, EMG, and respiration rate in office-like situations using various sounds as stressors. The induced stressors were validated through questionnaire answers. General Estimating Equations analysis was applied to classify the data into rest or stress state, achieving an accuracy of 74.5%. In [2], they developed an algorithm based on a multilayer perceptron (MLP), a Generalized Regression Neural Network (GRNN), and an Adaptive Network Based Fuzzy Inference System (ANFIS). They used the Stroop test combined with auditory stimuli (70 dB) obtaining 96.67% accuracy. However, the procedures are not described in detail. Sharma and Gedeon [48] combined GSR, ECG and blood pressure (BP) with eye gaze and pupil dilation (PD) to recognize stress. They asked the participants to read stressed and non-stressed types of texts, and they surveyed to confirm the stress states. They developed a hybrid genetic algorithm, which combined an Artificial Neural Network with an SVM, achieving 89% accuracy.

Other studies attempt to detect stress states and stress levels (low, medium and high). Healey and Picard [49] recorded ECG, EMG, GSR, and respiration rate, while drivers followed a predefined route in the Boston area. They concluded that GSR and HR are closely correlated with drivers’ stress levels, approaching an accuracy of 97%. De Santos Sierra et al. [50] used GSR, HR, and fuzzy logic to achieve the highest accuracy (99%) amongst the examined related studies. Their experiments focused on two stressing tasks, hyperventilation and talk preparation to give a speech in front of a recording camera. In another study, the researchers asked the participants to do arithmetic subtractions in configurable Virtual Reality (VR) environments while HRV, GSR, and ST were recording. After the experiment, they conducted two questionnaires surveys to evaluate the intensity of the stress. A Kernel-Based Extreme learning machine algorithm was used to classify stress achieving 95% accuracy [10]. Other researchers achieved an accuracy of 88.2% using drivers’ ECG measurements obtained from the MIT-BIH PhysioNet Multi-parameter Database and machine learning algorithms [51]. Another researcher employed la Stroop test and achieved 88.5% accuracy using GSR, ECG, EMG, and reaction time (RT) with machine learning algorithm [1]. In [52], they opted to record only EEG and classified the data through an SVM. They used the Stroop test and arithmetic test as stressors, reaching 75% accuracy. In another study [53], researchers asked the participants to do some tasks on the computers while some physiological signals (ECG, GSR, ST) were recording combined with behavioural and performance evidence. Through a Bayesian Network, they were able to infer user stress levels with 92% accuracy.

There are also researchers who aim to classify emotional states (stress, neutral, euphoria, anger, etc.). Picard et al. [25] developed such a method based on GSR, EMG, BVP, and respiration. They gathered data from one subject for more than six weeks. They used Clynes protocol to elicit emotions that sequences eight emotions, engages physical expression and prompts the subject to express the same emotion every three minutes. This study achieved the highest recognition accuracy (81%) on eight classes of emotions using the Fisher Projection. In another study, the researchers analysed the emotional state of four drivers in simulated race conditions by classifying vectors of features extracted from GSR, facial EMG, respiration, and ECG. The proposed system classified the emotional state using an SVM and was also able to detect the relative stress level of participants. Their achieved classification accuracy was 86% [54]. In [55], they tested linear and non-linear classifiers to classify emotions automatically using film clips. Participants were asked to self-report their emotional state after each film. They measured GSR, ECG, facial and eyeblink EMG, ST, and other physiological channels achieving an average classification accuracy of 84.5%. Kreibig et al. [56] distinguish fear, sadness, and calm, inducing films. Participants reported their perceived emotional state through a questionnaire. They recorded GSR, ECG, EMG, RESP, and other physiological measures and classified the emotions using pattern classification analysis, achieving 85% accuracy.

To summarize, various physiological signals can be used, alone or in combination, to detect stress. However, increasing the number of physiological signals does not necessarily ensure the highest possible accuracy. For instance, Wijsman et al. [47] tried to detect stress using four different signals, and their achieved accuracy was only 74.5%, while Setz et al. [44] used only GSR, and they achieved a higher accuracy (82.8%). However, Wijsman et al. confirmed the induced stressors carrying out questionnaire survey while Setz et al. did not. Besides the combination of physiological signals, the selected algorithms play a crucial role. Regarding the selected physiological signals, all researchers have used GSR, mainly combined with ECG or BVP. Methodologically, the use of SVM dominates previous studies for stress detection, as depicted in Table 1, but the highest accuracy has been achieved by an algorithm that combines MLP, GRNN, and ANFIS. However, details about the implemented algorithm are missing as well as the confirmation of the induced stressors by the participants. Concerning the studies for estimating stress levels, GSR and EMG were found to be most helpful. An introduced feature-based algorithm and fuzzy logic seem to contribute to more accurate stress level classification. Many researchers achieved accuracies over 95%. On the contrary, the achieved accuracy for emotional states classification does not exceed 90%. This is reasonable, as it is a generally complex task to measure distinct emotions, rather than general arousal or stress [25].

### 2.3. Stress Detection Using Wearable Sensors

An essential facilitator for physiological signal records is the use of new sensing capabilities dominating mainly in urban environments [57]. Most notably, wearable biosensors enable the continuous stream of physiological data with high temporal resolution [49] and can be used for basic research, clinical application, or during daily routines in real-life situations [58]. They are “objective” in that they do not require individuals to report on their current state and they are less interruptive because individuals can go about their regular routines. The rapid development of high-performance sensor technology has led to small and flexible wearable biosensors, which are the basis for pervasive sensing approaches [58,59]. These biosensors may be valuable tools to detect emotional or stress activation, as they provide high-quality data that are accurate, complete, relevant, timely, detailed, adequately portrayed, and retain adequate contextual information to support a decision-making process [60,61]. However, the use of wearable biosensors in real-world experiments poses several challenges in terms of reliable and useful measurements for emotion extraction [58]. First, the sampling frequency should be sufficient to depict the signal correctly. Second, the proper placement of the sensor is critical to avoid any ambiguities and to record the physiological signal accurately. Third, even if the sensor is properly placed, the raw physiological signals usually have a large number of small fluctuations caused by the oscillations of the physiological status of human bodies. These fluctuations are inevitably recorded. Thus, filtering the raw sensor signal is an essential task for noise removal to ensure stress detection with high accuracy [62,63]. Noise can be filtered by implementing various filters, such as the Kalman filter, Butterworth filter, Median filter, Wiener filter, or Wavelet Decomposition. The selection of the optimum filter depends on the nature of the signal, the features to be extracted, and the type of noise [35]. Last, it is not feasible for the researchers to control the environmental factors in real-world studies. Consequently, it is challenging to isolate the impact of a stimulus [58]. Despite all these challenges, a considerable number of studies have attempted stress detection using wearable physiological sensors, as presented in the previous section.

### 2.4. Research Gap

The literature review reveals plenty of methods for stress detection using wearable physiological sensors. The features that differ at each approach are (1) the use of different physiological signals, (2) the methodological approach, and (3) the design of the study (laboratory or real-world settings, induced stressor, and its confirmation). Concerning the physiological signals, various combinations have been investigated, as discussed in Section 2.2. As regards to the applied methods, machine-learning algorithms dominate, as the literature review revealed. With respect to study design, two categories of approaches have been pursued: The first uses laboratory data and does not investigate the efficiency of the method in real-world studies. This means that the proposed methods show limited validity outside the constraints of laboratory settings. Second, several approaches have been developed based on real-world data, but their validity for unambiguously detecting psychological stress is hampered by limitations inherent in defining the ground truth in the assessment of stressful events. Typically, previous approaches correlate physiological signals with the spatial characteristics of the physical environment, which is sometimes supported by video recordings. To our knowledge, there are a few research efforts that have aimed at (manually) combining the two approaches and define and detect stress in a well-controlled laboratory context and apply and extend the gained knowledge in real-world settings [14,15,16]. However, there is still a need for methodologies that can detect stress in real-world setting with high accuracy to better understand how people experience their urban environment. Besides this, previous studies (both lab studies and real-world studies) have mainly focused on stress detection while disregarding the stressors. Only a few studies confirm the stressors subjectively through interviews or objectively through videos.

## 3. Methodology and Laboratory Experiment

Our study is designed to bridge the gaps laid out above (methodology and study design). In a first step, we introduce a rule-based algorithm that combines expert knowledge with an empirical process to detect MOS using laboratory data. We further investigate the possibility to transfer the algorithm to real-world field studies through a mix-methods approach, which contributes to overcoming the issue of individually subjective perceptions.

### 3.1. Algorithm for the Detection of Moments of Stress

#### 3.1.1. From Bio-Sensing to Bio-Geodatabase

Our algorithm analyses GSR and ST (s. Section 3.2 for details) were recorded through a wearable, unobtrusive, and non-invasive wristband, the “Empatica E4”. This wearable has been designed for research and clinical purposes [64], including medical (FDA) and electronic certifications. The E4 has sensors to measure BVP, GSR, IBI, and ST in real-time. The sampling frequency of GSR is 4 Hz, the resolution is one digit 900 pico Siemens, and the range varies from 0.01 to 100 μS. The sampling frequency of ST in 4 Hz, the resolution is 0.02 °C, and the accuracy is ±0.2 °C within 36–39 °C. The device also contains a three-axis accelerometer to capture movement and is unobtrusive, allowing quick and easy use in studies under daily conditions.

In our study setup, the human sensor data were collected through an eDiary smartphone app, connecting the wearable sensor via Bluetooth. The eDiary app collects GSR and ST and the subjectively perceived stress levels and emotions. It creates a database containing the collected physiological signals and it automatically adds timestamps to all measurements based on the smartphone system time. It also geolocates the data using the phone’s built-in global navigation satellite system (GNSS) sensor, giving us the possibility to associate the stress response data with a spatial context in real-world experiments [65].

#### 3.1.2. Data Pre-Processing

We pre-processed the data with the following steps. First, we filtered the raw signals to reduce noise in the measurements. Thus, we applied a first-order Butterworth low-pass filter with a cut-off frequency of 5 Hz to remove high-frequency signal noise from GSR, induced through pressure on the device, body movements, irregular respiration, or device-internal technical reasons [66]. A low-pass filter with 5 Hz as the cut-off frequency filters out the noise and leaves GSR responses untouched [67]. Afterwards, we applied a first-order Butterworth high-pass filter with a cut-off frequency of 0.05 Hz to separate the SCR and the SCL of GSR, according to [66]. Concerning the ST, we used a second-order Butterworth low-pass filter with a cut-off frequency of 1 Hz and a second-order Butterworth high-pass filter with a cut-off frequency of 0.1 Hz. The selection of the filters and the associated cut-off frequencies was based on the findings of previous research efforts [11,68,69]. Second, we down sampled the physiological signals from 4.0 Hz to 1.0 Hz by calculating the average value of all the data falling into each 1-s window and cubic spline interpolation was used for boundaries. The aim was to obtain one value per second to establish comparability between the signals in the frequency domain. Third, we replaced the missing values with the average of the previous and next value from the dataset. This process was feasible since there were no two consecutive missing values. Thus, we acquired a bio-database, which contained EDA and ST measurements, sub-sampled to 1 Hz. Then, we assigned the smartphone’s location for every single second of the measurements. The outcome of the process above was the creation of a bio-geodatabase containing the used physiological signals (EDA and ST) associated with time and location. This bio-geodatabase was used to develop and calibrate the proposed algorithm.

#### 3.1.3. Algorithm Development

**Figure 1:**
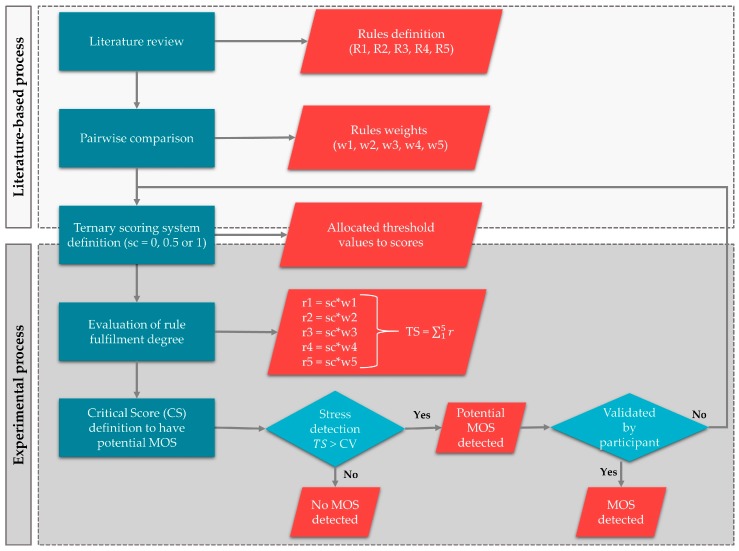
Methodology flowchart for the development of the algorithm for MOS detection.

We used the bio-geodatabase with the data from the laboratory experiment to develop a rule-based system. A rule-based system is a domain-specific expert system that uses rules to narrow down choices and can effectively automate problem-solving standards [70,71]. The aim was to detect the MOS that we induced during the laboratory experiment. Firstly, we defined some rules/criteria for our system and the crucial threshold values based on a literature review. Then, we assigned various weights to the rules based on the assumption that each rule has a different importance for stress detection. The allocating weights were the outcome of a pairwise comparison amongst the defined rules. This technique is widely used to deal with subjective and objective judgment about qualitative and quantitative criteria in multi-criteria decision making [72]. Finally, we adopted a ternary scoring system (0, 0.5 and 1) to assess the degree of rule fulfilment (not at all, partially, and fully). We determined critical values to assess the partial fulfilment through an experimental process; modifying the critical values to successfully detect the maximum possible number of induced MOS. This process was challenging and time-consuming, so we decided not to use more scores to evaluate the degree of fulfilment. The whole process is schematically presented in Figure 1.

**Figure 2:**
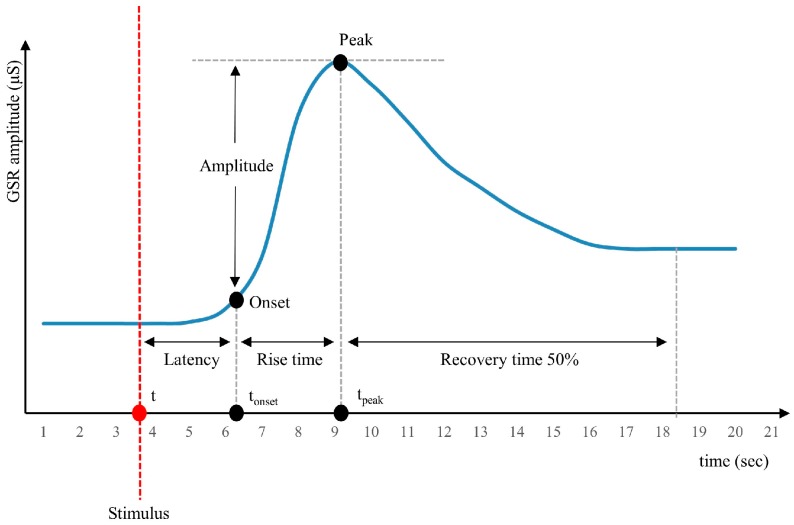
Schematic GSR to a hypothetical stimulus.

Figure 2 presents the characteristics of GSR response to a hypothetical stimulus used to define the rules as below:

Let t designate the time of an acute stressor. Let g represent the GSR and T the ST.

R1—GSR Amplitude Increase. A GSR increase for 2 to 5 consecutive seconds, in-between a local minimum and a local maximum GSR value, is the first indication to detect a MOS. As shown in Figure 2, GSR increases seconds after the stimulus. Therefore, if no change is detected within 5 s of stimulus onset, it is assumed that no measurable response has occurred [20]. Thus, the first derivation should be positive for 2 to 5 successive seconds. So, if Equation (1) is satisfied for the second t, then it is assigned the maximum score “1”.

[g_t_:g_t+5_]′ > 0, (1)

The rising time of GSR decreases as the stimuli increases [73]. This implies that if the duration of GSR is greater, the stimulus is not intense. Based on this, we defined that if the GSR derivative is positive for more than 5 s, the score “0.5” is given. Otherwise, it is assigned the score “0”.

R2—ST Decrease. To have a candidate MOS, it is required that ST decreases 3 s after the GSR increase [65]. We defined 3 s as the minimum duration of ST decrease. Thus, if the Equation (2), which stands for this rule:

[T_t+3_:T_t+m_]′ < 0, (2)

where m ≥ 3, is fulfilled, the score “1” is allocated. During algorithm calibration, we observed that an ST decrease might start from 2 to 6 s after the increase of GSR. In this case, the candidate MOS is scored with “0.5”.

R3—Rising Time. According to literature, this may vary from 1 to 5 s, as it has been aforementioned. Thus, the time difference between a local minimum and a local maximum will be less than 5 s to have a potential MOS. However, during the experimental phase, it was noticed that there were rising times of more than 5 s. In that case, the score “0.5” is assigned instead of “1”.

t_peak_ − t_onset_ ≤ 5 s (3)

R4—Response Slope. This feature combines the amplitude with the rising time or GSR; according to previous research, steeper slopes are associated with more stressful events [31,32]. Thus, it is required that the slope will exceed the critical threshold value of 10°. The experimental phase revealed that potential MOS may have a slope greater than 8°. For this case, the score “0.5” is assigned.

(g_peak_ − g_onset_)/(tg_peak_ − tg_onset_) ≥ 10° (4)

R5—Duration. The last rule is an assumption that it is feasible to have only one MOS in a time window of 10 s. This assumption is related to the typical values of latency, rise time and half recovery time (1–5, 1–5, 1–10, respectively). We used the mean values, and we argued that a stress event has an average duration of 10 s. This argument implies that if a MOS is detected at time t, it is not feasible to detect another from t to t + 10.

t_MOSi+1_ − t_MOSi_ > 10 s (5)

We initialized the proposed algorithm with the first experimental critical values for partial satisfaction. Every second of measurements was examined based on the abovementioned rules and scored according to the degree of rule satisfaction. Thus, every second had a score associated with every single rule. Then, this score was multiplied by the weight of importance of each rule respectively. The following equation summarises this procedure:

r_n_ = sc*w_n_ (6)

where sc is the given score for the rule and w_n_ is the associated importance weight of rule n and $\sum_{1}^{5}w_{n}=100$. The Total Score (TS) for a second is calculated by:

(7) $$\mathrm{TS}=\sum_{1}^{5}r$$

The maximum achievable TS is 100, provided that all rules are scored with “1”, so it follows that 0 ≤ TS ≤ 100. A stress response may vary in terms of intensity and characteristics. Aiming to integrate this feature at our algorithm, we did not choose 100 as a critical score. Instead, we defined 75 as the Critical Score (CS) to have a potential MOS based on empirical evidence from our lab studies. Therefore, if the Equation (8) is satisfied, a MOS is detected:

TS ≥ 75
(8)

The definition of critical values was challenging. The aim was to achieve the optimum calibration for our algorithm. This was achieved through an iterative process, as it is shown in Figure 1; modifying the critical values to maximise the number of detected induced MOS using the laboratory data as calibration data. Even a small change may significantly affect the MOS detection, which necessitated the investigation of several parameter settings, resulting in an optimum combination for our algorithm. The final critical values are portrayed in Table 2 and they are tailored to detect a potential MOS using the sensors, the methods and the data that have been described in Section 3.1.1 and Section 3.1.2. These values cannot work as global critical values for MOS detection.

### 3.2. Laboratory Experiment

For the purpose of the current study, we carried out a laboratory experiment. We induced auditory stimuli (s. “Experimental Protocol” below), while the physiological signals of subjects were recorded through wearable sensors. This experiment aimed to assign the physiological responses to each stressor, i.e., to generate a “gold standard”, and to further develop an algorithm for stress detection. Laboratory emotion elicitation works reliably and induces important aspects of emotion. The constrained environment permits eliciting, controlling, and measuring an emotional response reliably [12].

#### 3.2.1. Subjects

We invited participants through personal e-mails and finally we recruited nineteen subjects; 11 women and 8 men with various cultural backgrounds. According to literature, women show stronger responses to a stressor than men [74]. However, the sample was too small to investigate this difference. All participants were healthy without any muscle or heart disease or mental disorders based on the questionnaire responses (s. Q1 in the experimental protocol below). Their age ranged from 25 to 45 with various cultural backgrounds. All participants were non-smokers and they had been advised to avoid alcohol or caffeine consumption in the morning before the study. Participants were aware of the aim of the research but not of stressor details. The study had been approved by the local ethics committee and informed consent was obtained from all subjects before participation.

#### 3.2.2. Experimental Protocol

The protocol of the laboratory study was tailored to induce stress reactions. The test was performed using a laptop in a quiet room with the least possible distractions. In the initialization phase of the protocol, there was a rest period of 5 min to calibrate data and establish an individual stress profile. Participants were sitting on chairs not facing each other. They were instructed to avoid any cognitive and physical activity, to relax, and to prevent interaction amongst each other. In the stress phase, subjects were exposed ten times to the same auditory stimulus. The sound was played through speakers and it was the sound of an air-horn. Thus, we induced ten moments of stress in total at every session at pre-determined times. All the auditory stimuli had the same characteristics, same duration (1 s) and the same intensity. We selected this duration based on the following principles: (1) The auditory reaction time is faster than the visual reaction time [75], so we can have a short stimulus. (2) We hypothesize that if the subjects hear the same sound for many seconds, they will sooner become tolerant to the stimulus; this was not our aim. The time interval between the stressors was random, but at least 60 s to avoid overlapping responses. Another resting phase of 5 min followed this stress phase. Two questionnaires were involved in the protocol: the first one (Q1) was given to the participants before the experiment and asked about the general condition of their health, alcohol and caffeine consumption the last 24 h and medication use. In the second questionnaire (Q2), subjects were asked to note down their perceived stress intensity after each affective stimulus using a 5-point scale (“calm—no stress”, “low stress”, “moderate stress”, “high stress”, and “extreme stress”).

### 3.3. Algorithm Validation in Real-World Settings

As described above, our aim was to develop an algorithm that is transferable to real-world settings. The proposed algorithm was calibrated using laboratory data, which can be affected by intrinsic factors such as pain or fatigue. However, the transfer to real-world settings may be constrained because real-world data may be affected by both intrinsic and extrinsic factors (weather, humidity, light, etc.). Thus, we used a multi-disciplinary mixed-methods approach to overcome this shortcoming: we combined spatial analysis of detected MOS from physiological measurements with an eDiary app, ego-perspective videos, and personal interviews. Ego videos permit to overcome the constraint of subjectivity and bias that may be induced by participants’ individual perceptions. Thus, we were able to associate the geo-located MOS with actual stressful events and self-reported stress. We used the following methods to assess the performance of our algorithm in real-world field studies and to further validate the transferability from lab settings to real-world settings:

1. Spatial analysis of MOS: a spatial analysis of the geo-located MOS was performed to identify spatial patterns in urban areas. We used a MOS ratio to standardize the data. This method is described with detail in [76]. Then we performed hotspots analysis, which based on Tobler’s first law of geography “Everything is related to everything else, but near things are more related than distant things” [77]. This low is quantified by the presence of significant spatial autocorrelation [78]. Thus, we perform a hot spot analysis by using the Gi* method [79] using the following equation:
  (9) $$G_{i}^{*}\left(d\right)=\frac{\sum_{j\neq 0}^{n}w_{ij}x_{j}-\;W_{i}^{*}\bar{x}}{SD\left(x\right){\left\{\left[\left(nS_{1i}^{*}\right)-W_{i}^{*}{}^{2}\right]/\left(n-1\right)\right\}}^{\frac{1}{2}}}$$
  where $S_{1i}^{*}=\sum_{j=1}^{n}w_{ij}^{2}$ and $W_{i}^{*}=\sum_{j}^{n}w_{ij}.$

*W_ij_* represents the spatial weight shared by points *i* and *j*, and *x* represents the variable value for location *j*. Significance testing with Gi* can also be done by using a normal approximation. The statistic, as presented at the above equation, is already in the form of a z-score, so no further conversion is required. The outcome of the spatial analysis was a map with hot spot areas that denote stressful areas and cold spot areas which denote areas of relaxation.

2. eDiary: we introduced the eDiary app and we asked participants to input their perceived emotional state by pressing a button on the smartphone’s screen and input their perceived emotions during the trips. The eDiary app is described in Section 3.1.1. The collected data were geo-located point data with emotional state description (stress or calm) able to be projected on maps.
3. Ego-perspective videos: we mounted cameras on participants’ chests to record the trips. This permits us to visually identify the real-world situations which worked as objective emotional triggers and correlate them with the detected MOS from our algorithm.
4. Personal interviews: after the completion of one trip, the participants were asked to describe any stressful event that they experienced through an additional questionnaire survey. Privacy issues did not arise as the questions were as general as possible without asking sensitive topics to avoid putting them in an awkward situation. Participants’ concerns were also acquired.

## 4. Results

**Figure 3:**
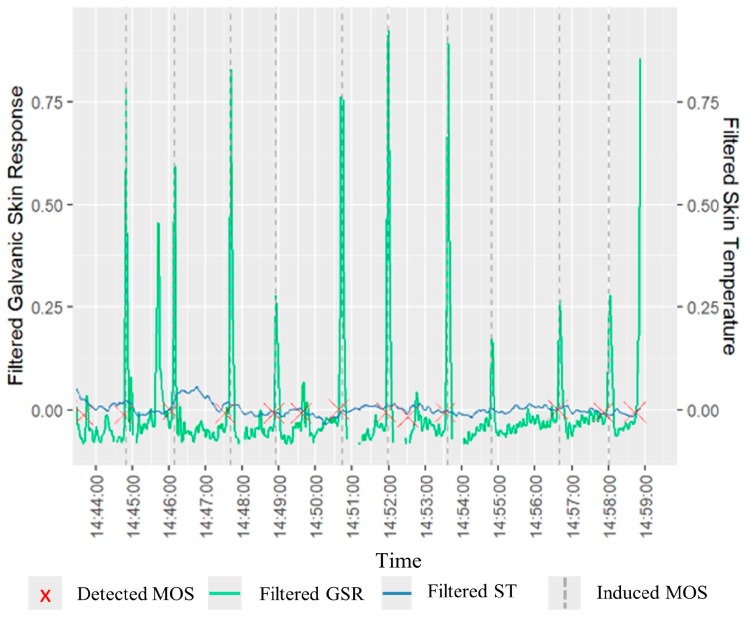
A typical example of a time series plot for a participant.

In the design phase of our algorithm, we carried out a laboratory experiment (s. Section 3.2). Figure 3 shows the resulting time series plot of one participant, where the induced MOS (air horn) are shown in vertical dashed lines, GSR is illustrated in green, ST in blue, and the MOS detected by our algorithm are depicted by red crosses. In this example, the algorithm detected nine induced MOS (out of 10 induced MOS), and the participant perceived 10 MOS according to their self-reported stress. However, in many cases, the algorithm detected MOS at times where we did not induce any stimuli. For instance, for the same participant, the algorithm detected four MOS additionally to the induced ones as it is shown at the same figure. At the end of the calibration phase, the algorithm detected on average 6.9 True Positive MOS (TP MOS) per participant and 3.9 MOS, whereby participant may have felt stress additionally to our artificially induced ones, which we verified through a self-reporting assessment (s. below).

**Figure 4:**
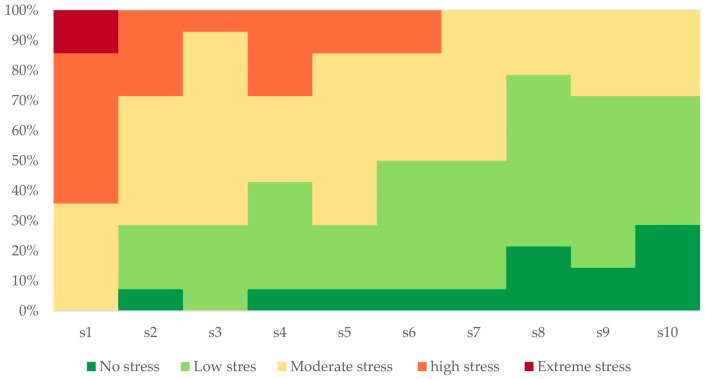
Participants’ self-report perceived stress for ten stressors.

The self-reported assessment of perceived stress intensity for each of the ten stressors provided valuable information about the participant’s subjective stress levels. We used these results to validate the induced stressors. Figure 4 shows the self-reported stress levels of all the participants from the laboratory experiment. More than 70% of the participants felt stress in response to all the stressors, while 90% of them felt stress at least seven times. The perceived stress levels for the first induced stressor ranged from “Moderate stress” to “Extreme stress”. Stressors 3 to 7 elicited, mainly moderate- and low-stress levels, whereas stressors 8 to 10 induced mostly low-stress levels. This concurs with the phenomenon of habituation, describing the reduction of the physiological responses elicited by repeated exposure to a monotypic stressor [80]. The percentage of participants who stated “no stress” is less than 10% for most of the stressors. However, we notice that this percentage increases to 20% for the last stressor.

During the calibration phase, we made the following significant observations:

- O1—True Positives MOS (TP MOS). The algorithm was able to detect 6.9 TP MOS on average per participant while the participants perceived 8.5 MOS on average.
- O2—False positive MOS (FP MOS). The algorithm detected MOS at times where we did not induce any stimuli. We could associate the FP MOS with participants’ feedback: “I didn’t know when to expect the next stressor, and this stressed me” or “When we did not hear any stimuli I thought that I have such a busy day and this made me feel stressed”. FP MOS could also be associated with other intrinsic reasons which provoke stress, such as pain or fatigue [4]. Additionally, according to pathophysiology, an infection can cause the body’s temperature to rise internally, and several mechanisms can cause body temperature to rise externally. Thus, we cannot exclude the possibility of variations at skin temperature that led to false positive MOS cause of fever states even though we recruited healthy participants and we did not find indications for fever [81].
- O3—Discordance between self-report stress and physiology indicators. The algorithm detected MOS at times when we induced a stimulus, but the participants perceived “no stress” based on the questionnaire responses.

## 5. Real-World Field Studies

The outcome of the proposed methodological approach is an algorithm that detects MOS and associates them with time and space. Thus, the implementation of the algorithm allows identifying when and where a participant is stressed. We applied our physiological stress detection algorithm to various field data and evaluated its efficiency during real-life events using a mixed-methods approach that is described in Section 3.3 (eDiary entries, ego videos and personal interviews).

### 5.1. Multi-Purpose Bike Lane and Cyclists’ Safety

**Figure 5:**
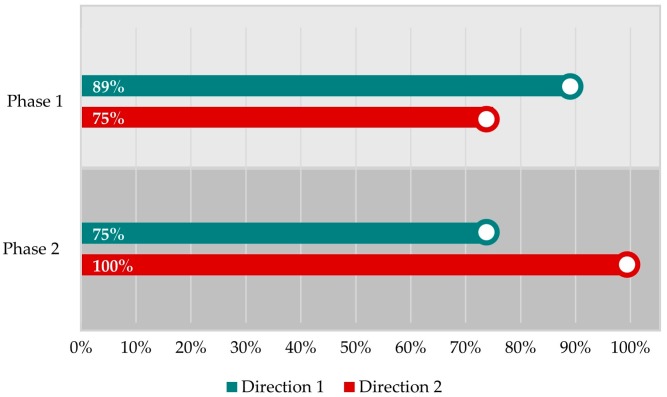
Validation of detected “stressful” areas by the mixed-methods approach.

**Figure 6:**
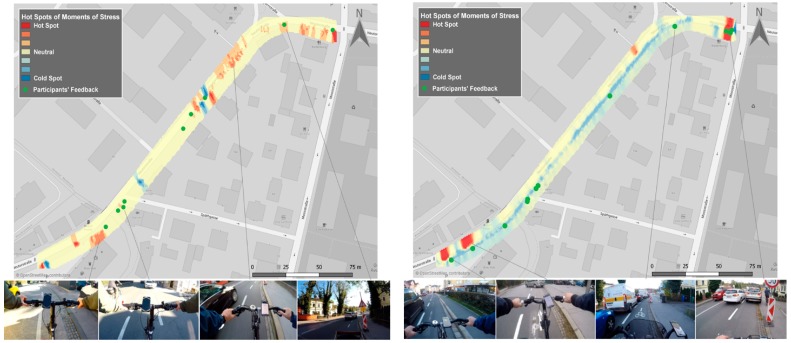
Hotspot maps of detected MOS, phase 1 (on the left), and phase 2 (on the right), direction to the city.

**Figure 7:**
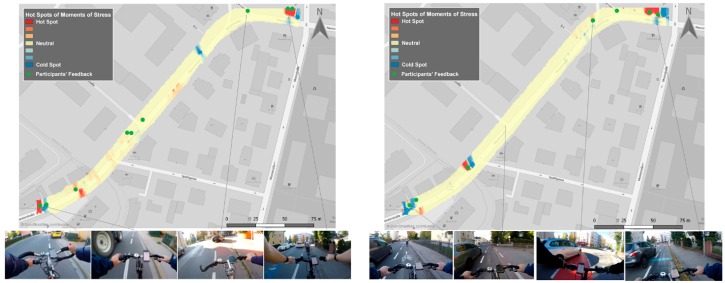
Hotspot maps of detected MOS, phase 1 (on the left), and phase 2 (on the right), direction from the city.

The first study took place in the city of Salzburg, Austria, in collaboration with a pilot project of the municipality. The pilot project aimed to investigate whether a wider multi-purpose bike lane would improve cyclists’ safety. In the first phase of the study (30 October 2018), 12 participants cycled a predefined path in both directions (direction 1: to the city center and direction 2: from the city center) at bike lanes with 1.3 m width. In the second phase (30 November 2018), the same participants followed the same path at the same time slot under similar weather conditions, but the bike lanes had wider widths varying from 1.75 to 2 m. We collected and analyzed human sensor data to probe the cyclists’ emotional state for both phases. The participants were recruited and equipped with wearable sensors, action cameras and smartphones. Figure 5 shows the results of our analysis combining the data of all participants for both phases and both directions. The revealed hotspots are shown in Figure 6 and Figure 7 indicate the most “stressful” (red) and “relaxed” (blue) areas. The detected MOS and reported MOS layers (eDiary inputs and interviews) correspond well spatially, while video tracks confirm this connection. Based on the interviews and the video tracks, the most common triggers are cars passing close by, long waiting times, parked cars on bike lanes and construction areas. Moreover, 89% of hotspots could be verified using participants’ feedback (eDiary inputs and interviews) for direction 1 (to the city) in the first phase. In the second phase, 75% of all MOS could be verified for the same direction and second phase. For direction 2 (from the city), 75% of “stressful” areas were validated in the first phase and 100% in the second phase.

Figure 6 and Figure 7 present the detected “stressful” areas as hotspots and the correlated objective emotional triggers identified through the ego-perspective videos. It is obvious that for both directions, the widened multi-purpose bike lane improved cyclists’ perceived safety since there are fewer hotspots. More precisely, the algorithm detected 20% fewer MOS in the second phase with the same number of participants and rides. The algorithm detected MOS that could not be related to a stressful event, but there were no hotspots that could not be validated by participants’ feedback.

### 5.2. Urban Walkability

**Figure 8:**
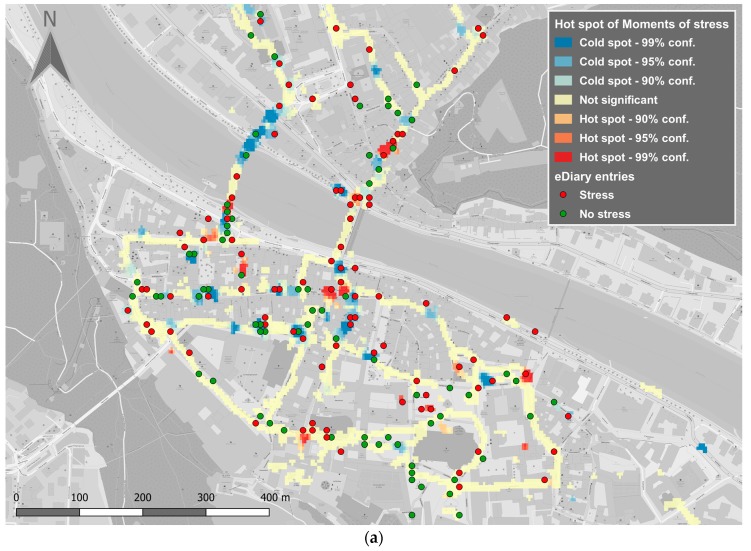

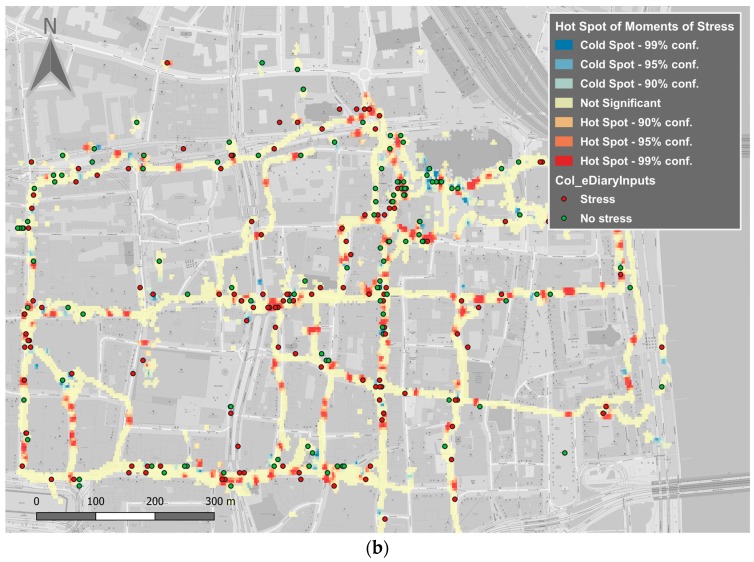
Hotspots of pedestrians’ MOS in different cities: (**a**) Salzburg; (**b**) Cologne.

In another field study, we investigated the urban walkability in the city of Salzburg, Austria and the city of Cologne, Germany. We recruited 56 participants (27 for Salzburg and 29 for Cologne) who were instructed to walk through their respective cities with sensors mounted on their bodies (Empatica e4 wristband, Zephyr BioHarness3, plus GoPro ego-video camera). They were also asked to enter inputs into the eDiary app on a smartphone that they carried with them and to answer a customized questionnaire after their walk. Figure 8 shows the Hotspot analysis (Getis Ord Gi*) of physiological sensor data for the city of Salzburg, whereby red areas indicate hot spots (spatially clustered moments of stress) and blue areas indicate cold spots (spatially clustered moments of relaxation). Generally speaking, the results show that both hot spots and cold spots identified in the human sensor data correlate with the subjective perceptions of the participants provided through the eDiary app and the questionnaire. This correlation is similar in both test cities. More details on the qualitative conclusions can be found in [82].

### 5.3. Urban Wellbeing

**Figure 9:**
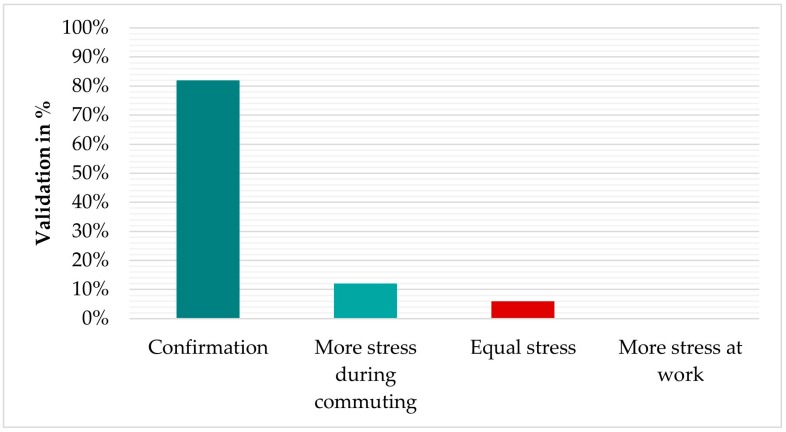
Validation of self-reported stress by the algorithm’s results.

Another field study was carried out in collaboration with the University of Birmingham to investigate the urban wellbeing based on self-reported MOS through interviews and eDiary app entries and detected MOS from our algorithm. The research took place in Salzburg, Austria and Birmingham, UK. In these studies, 31 participants were recruited (9 and 22, respectively) for the study and they were asked to wear the physiological sensors continuously for a whole day, including (1) the way from home to work, (2) during working hours, and (3) the way from work back home. Then, the subjects reported their perceived stress through personal interviews. Figure 9 presents the results of the comparative analysis between the perceived and detected MOS. We were able to confirm 82% of the self-reported stress events based on the detected MOS. The algorithm also indicated that sometimes, participants were more stressed at work than they reported, based on the number of detected MOS, or they had equal stress between commuting and work. Psychometric responses may be affected by participants’ mood during the study and this could be a reason for the difference between the perceived emotional state and the actual state. Besides, it is possible that participants may not accurately answer how they feel. Instead, they may answer in a way they believe will satisfy the researchers [83].

## 6. Discussion

### 6.1. Developing a Stress Detection Algorithm

The proposed algorithm is able to detect 6.9 induced MOS on average, from GSR and ST measurements, while the average number of self-reported MOS is 8.5. However, self-report may be affected by a bias, which induced from participants’ individual perceptions. The algorithm also detected 3.9 MOS on average additional MOS to the induced ones at times where we did not induce any stimuli. Nevertheless, the interviews indicated that the participants felt stressed at times between the stimuli induce. Finally, yet importantly, the algorithm confirmed the discordance between self-report stress and physiological indicators, detecting induced MOS, which have been perceived as “no-stress” by the participants.

The global average accuracy of the proposed methodology in real-world settings is 84%. We tested the algorithm with both cyclists and pedestrians, and the congruence between detected MOS and self-reported stress (eDiary entries and interviews) and recorded stress (ego video) was high. This is an indication that the proposed algorithm works reliably, but the accuracy is still improvable. Many studies have achieved higher accuracies, but most of them took place in laboratory settings and their performance in real-world field studies is unknown. Besides that, only a few studies confirmed the stressors objectively or subjectively. This makes our algorithm outperform studies that have achieved lower accuracy in laboratory settings [47,52] and studies that have higher accuracy in laboratory settings without confirming the stressful moments. Moreover, most of the researchers who have achieved similar or higher accuracies, mainly in laboratory settings, have used more physiological signals [25,44,46] while we use only GSR and ST making our approach excelling in terms of data collection requirements. The accuracy was reasonably high using only two physiological signals and this could be because both signals indicate sympathetic activation. Thus, the sympathetic output from the hypothalamus is measured at two different endpoints that are controlled by the sympathetic nervous system. Our algorithm is looking for activation of physiological signals in different time windows to detect stress but requires an activation match; a temporally shifted coincidence. This excludes parameters, such as motion or electronic artefacts that could deteriorate the physiological signals at the same time and the accuracy.

Motion is a crucial parameter that could degrade the quality of measurements. A physical exertional response modulates the activity of the ANS [84] as it activates rather the same physiological processes as emotional or stress activation [12]. Healey and Picard [49] achieved 97% accuracy, introducing a feature-based algorithm to detect emotions during real-world driving tasks using more physiological signals. However, at their experiment, the participants were driving without having any physical exertion while in our real-world field studies, the participants were freely moving. Future work will focus on integrating patterns of exertion state into the algorithm to reassure that we detect mental stress instead of physical activation.

Caution needs to be taken in generalizing from a relatively small sample of participants since a vast literature of stress research demonstrates how stress responses can be affected by social characteristics such as gender, ethnicity, age, relative income, adverse childhood experiences, and upbringing. We recruited 19 participants aged from 25 to 45 years old and we calibrated the algorithm based on these data. Therefore, the generalization to the entire population at all age ranges may lead to unreliable results. However, in the real-world field studies, we recruited different participants with a greater variance at the age and the correlation between detected stress areas and self-reported stress was high. A logical next step is to acquire more data to calibrate the algorithm through a laboratory experiment recruiting participants with greater diversity in age. Thus, it will be feasible to generalize the algorithm to the entire population with fewer ambiguities concerning the parameter of age. Another important aspect is that the algorithm provides a binary classification of stress (stress or not). However, people may experience stress at multiple strengths [16]. Thus, another future research task is the incorporation of multiple levels of stress to produce more fine-grained results.

### 6.2. Laboratory Versus Real-World Settings

Although our algorithm produced reliable results and outperformed previous approaches, there are some limitations concerning the transferability of our laboratory-based algorithm to real-world settings. One significant divergence is that in the laboratory studies, the conditions are described to the participants, while, in the real-world, the subjects have natural preferences and choices about the situations they seek and avoid. Another important aspect is that we induced the same short auditory stimulus 10 times in our laboratory experiment. Therefore, our stimulus may not elicit equally intense stress responses over time and the repetition may have led to experimental habituation based on MOS detection and self-reported assessment. This shortcoming may be mitigated by inducing different stressors in the laboratory study.

On the contrary, real-life stressors may be much more intense than ethically prescribed laboratory stressors, and the human tendency for the avoidance of stressful situations in real-life may make it difficult to find sufficient stressful and/or emotional episodes in a typical daily routine [12].

Another critical aspect is that the stress detection in real-life may be less accurate because of the unrestricted movement of participants and improperly worn devices. In laboratory experiments, movements and activities are limited, and the researchers have the chance to intervene [60]. Thus, it is possible to have increased motion artefacts and reduced performance. The integration of activity recognition schemes into the algorithm may mitigate this issue.

Finally, at the laboratory experiment’s protocol foresaw a resting phase of 5 min in the (it is possible that some participants will require a longer time to physiologically accommodate the experimental environment). During the resting phase, the participants performed no physical activity in order to best possibly calibrate the measurements to their individual physical condition. This calibration is more challenging in real-world settings where the participants are free to move.

Despite these limitations, our data indicate that traffic situations elicit sufficient variance in stress response to reliably detect high accordance between physiologically identified, self-reported, and videotaped moments of stress.

## 7. Conclusions

This paper presents a novel algorithm for detecting moments of stress through a rule-based algorithm combining galvanic skin response and skin temperature measured by low-cost wearable physiological sensors. We developed a framework of rules with associated weights and critical values for stress detection through literature and experimental process. We then carried out a laboratory experiment inducing auditory stimuli to generate a “gold standard” of physiological responses to stressors and to further calibrate the algorithm. We confirmed the induced stress through a questionnaire survey, which revealed that 90% of subjects had a stress response to almost all stimuli. At the end of the calibration phase, the algorithm was able to detect a maximum of 90% of the induced MOS and 77% on average. In addition, the algorithm detected induced MOS that had been not considered as stress by the participants. However, spikes in the physiological signals indicated a different state.

Then, we used the spatiotemporal module of the algorithm, and we carried out real-world field studies to investigate the efficiency of the proposed approach. Self-reported stress, from both eDiary and interviews, was used to ground-truth our detected MOS to ensure reliability [15,85,86]. Additionally, video tracks were used to validate the detected MOS in the context of the physical environment of city traffic.

Our results overall demonstrate that stress can be reliably detected with an accuracy that varies from 75 to 100%, with an average of 84%. However, there were detected MOS that could not be validated by the video tracks or the interviews. This could be false positives or a physiological stress state not recognized by participants. Self-report can be shaped by many social factors, leading to discordance between physiological and self-reported stress or emotion measures. Further, stress physiology in traffic situations may be elicited by experienced fatigue, pain, disease or environmental pollutants [4], i.e., events or mental states that were not systematically assessed.

In conclusion, it can be stated that the research presented in this paper can enhance the detection of stress states in real life and may thus foster a better understanding of circumstances that bring about physiological stress in humans. The framework of rules we have developed inform strategies to further improve stress detection, but the defined critical values are tailored to the current research, they do not serve as global values for stress detection. Furthermore, the automated means for recognizing a MOS in space and time provides an integrated methodology that is transferable to a variety of real-world applications of high societal importance. The validation of detected MOS, either with video tracks or with participants’ feedback, contributes to better understanding daily stressors. This extracted information brings the enormous potential for a variety of applications, particularly in urban areas where there are various urban stressors (traffic noise, da8889ngerous driving situations, fear of crime, crowding), and it is crucial for human wellbeing that these stressors are decreased [87,88]. Stress may be socially private information for some and participatory sensing applications such as the eDiary app collect information that is private, sensitive or confidential. Therefore, privacy-preserving steps have to be taken into consideration [86].

There are several open challenges, which will be addressed in future research, including the optimization of the algorithm integrating HRV, exertion states, activity schemes, improving the experimental protocol, complementing with machine learning algorithms, including a wider diversity of research participants, and using multiple levels of stress detection.

## Figures and Tables

**Table 1:** Summary of Literature Review.

Studies	Physiological Signals	Other	Method	Settings	Stressor	Confirmed Stressors	Accuracy
**Stress Detection**							
Setz et al. (2010)	GSR		SVM	Lab	Social-evaluation of arithmetic problems to be solved under time pressure	No	82.8%
Zhai and Barreto (2006)	GSR, ST, ECG, BVP	Pupil dilation	SVM	Lab	Stroop Test	No	90.1%
Hosseini and Khalilzadeh (2010)	GSR, BVP, RESP, EEG		SVM	Lab	IAPS pictures	No	82.7%
Wijsman et al. (2013)	GSR, ECG, EMG, RESP		General Estimating Equations	Lab	Auditory	Yes	74.5%
Lee et al. (2004)	GSR, ST, ECG		Introduced algorithm combining MLP, GRNN and ANFIS	Lab	Stroop test and auditory stimuli	No	96.7%
Sharma and Gedeon (2013)	GSR, ECG, BVP	Pupil dilation, eye gaze	ANN with SVM	Lab	Read stressed and non-stressed types of texts	Yes	89%
**Stress Detection and Associated Level**							
Healey and Picard (2005)	GSR, ECG, EMG, RESP		Feature-based algorithm	RW	Driving task	Yes	97%
de Santos Sierra et al. (2011)	ECG		Fuzzy logic	Lab	Hyperventilation and Talk Preparation	No	99.5%
Cho et al. (2017)	GSR		Kernel-based Extreme learning machine algorithms	Lab	Arithmetic subtractions in configurable Virtual Reality	No	95%
Keshan, Parimi, and Bichindaritz (2015)	EEG		Random Tree	-	-	No	88.2%
Zhang (2018)	GSR, ECG, EMG	Reaction time	SVM	Lab	Stroop test and auditory stimuli	No	88.5%
Jun and Smitha (2016)	EEG		SVM	Lab	Stroop test and mental arithmetic test	No	75%
Liao et al. (2005)	GSR, ST, ECG	Finger pressure, visual features	Bayesian Network	Lab	Tasks on the computer	No	92%
**Emotional States Classification**							
Picard et al. (2001)	GSR, EMG, BVP, RESP		Feature-based algorithm	Lab	Clynes protocol	No	81%
Katsis, Ganiatsas, and Fotiadis (2006)	GSR, EMG, RESP		SVM	Lab	Simulated race conditions	No	86%
Kolodyazhniy, Kreibig, Gross, Roth and Wilhelm (2011)	GSR, ECG, EMG, ST	Capnography, Piezo-electric sensor, plethysmography	KNN	Lab	Films	Yes	84.5%
Kreibig, Wilhelm, Roth and Gross	GSR, ECG, EMG, RESP	T-wave amplitude, Systolic and diastolic arterial pressure, HRV, Pulse wave amplitude at the ear	Pattern classification analysis	Lab	Films	Yes	85%

Abbreviations: GSR: Galvanic skin response; ST: Skin temperature; ECG: Electrocardiogram; BVP: Blood Volume Pulse; RESP: Respiration; EEG: Electroencephalogram; SVM: Support Vector Machine; MLP: Multilayer Perceptron; GRNN: Generalized Regression Neural Network; ANFIS: Adaptive Network Based Fuzzy Inference System; ANN: Artificial Neural Networks; Lab: Laboratory; RW: Real-world; KNN: k-nearest neighbors algorithm; HRV: Heart Rate Variability.

**Table 2:** Framework for stress detection: rules, critical values, and the adopted ternary scoring system.

Rule	Phys. Signal	Feature	Condition for Scoring Value: 1	Condition for Scoring Value: 0.5	Condition for Scoring Value: 0
R1	GSR	Increase	[g_t_:g_t+n_]′ > 0 where 2 ≤ n ≤ 5	[g_t_:g_t+n_]′ > 0 where 5 < n ≤ 8	[g_t_:g_t+n_]′ > 0 where n < 2 and n > 8
R2	ST	Decrease	[T_t+3_:T_t+m_]′ < 0 where m > 3	[T_t+2_:T_t+m_]′ < 0 where 5 ≤ m ≤ 6	[T_t+3_:T_t+m_]′ < 0 where m < 3
R3	GSR	Rising time (RT)	$1\leq t_{\mathrm{peak}}-t_{\mathrm{onset}}\leq 5$	$5<t_{\mathrm{peak}}-t_{\mathrm{onset}}\leq 15$	$t_{\mathrm{peak}}-t_{\mathrm{onset}}>15$
R4	GSR	Response slope (RS)	$\frac{g_{\mathrm{peak}}-g_{\mathrm{onset}}}{t_{g_{\mathrm{peak}}}-t_{g_{\mathrm{onset}}}}$ ≥ 10°	$\frac{g_{\mathrm{peak}}-g_{\mathrm{onset}}}{t_{g_{\mathrm{peak}}}-t_{g_{\mathrm{onset}}}}$ ≥ 8°	$\frac{g_{\mathrm{peak}}-g_{\mathrm{onset}}}{t_{g_{\mathrm{peak}}}-t_{g_{\mathrm{onset}}}}$ < 8°
R5	-	Δt between MOSi and MOSi + 1	$t_{{\mathrm{MOS}}_{i+1}}$ − $t_{{\mathrm{MOS}}_{i}}\leq$ 10 s	-	$t_{{\mathrm{MOS}}_{i+1}}$ − $t_{{\mathrm{MOS}}_{i}}$ > 10 s

Abbreviations: GSR: Galvanic skin response; ST: Skin temperature; MOS: Moment of stress.
